# Application of data mining techniques to explore predictors of upper urinary tract damage in patients with neurogenic bladder

**DOI:** 10.1590/1414-431X20176638

**Published:** 2017-08-17

**Authors:** H. Fang, B. Lu, X. Wang, L. Zheng, K. Sun, W. Cai

**Affiliations:** 1 Shenzhen Hospital, Southern Medical University, Shenzhen, China; 2 The Third Affiliated Hospital of Sun Yat-Sen University, Guangzhou, China

**Keywords:** Neurogenic bladder, Upper urinary tract damage, Decision tree model, Urodynamics, Urethra function

## Abstract

This study proposed a decision tree model to screen upper urinary tract damage (UUTD) for patients with neurogenic bladder (NGB). Thirty-four NGB patients with UUTD were recruited in the case group, while 78 without UUTD were included in the control group. A decision tree method, classification and regression tree (CART), was then applied to develop the model in which UUTD was used as a dependent variable and history of urinary tract infections, bladder management, conservative treatment, and urodynamic findings were used as independent variables. The urethra function factor was found to be the primary screening information of patients and treated as the root node of the tree; Pabd max (maximum abdominal pressure, >14 cmH_2_O), Pves max (maximum intravesical pressure, ≤89 cmH_2_O), and gender (female) were also variables associated with UUTD. The accuracy of the proposed model was 84.8%, and the area under curve was 0.901 (95%CI=0.844-0.958), suggesting that the decision tree model might provide a new and convenient way to screen UUTD for NGB patients in both undeveloped and developing areas.

## Introduction

Patients with neurogenic bladder (NGB) not only suffer from the inconvenience caused by lower urinary tract symptoms, e.g., urinary incontinence and retention, but also have the risk of upper urinary tract damage (UUTD) including vesicoureteral reflux, ureteral dilation, and hydronephrosis (1). The principal aims of treatment to NGB patients are preservation of the upper urinary tract function, avoidance of the risk factors, prevention of upper urinary tract complications, and maximization of patients' life expectancy. Currently, considerable morbidity (20–80%) of UUTD in NGB patients has been reported in a variety of studies (2).

UUTD is one of the most severe complications of NGB; therefore, early diagnosis is very important for a timely neurological management to avoid any further damage. Generally, upper urinary tract damage is detected by cystourethrogram, video urodynamic studies, or other image examinations. However, in many undeveloped or developing areas, it is not easy for patients to get access to these examinations conveniently due to economic reasons or equipment shortage. Therefore, a more convenient and economical method to predict the risk of UUTD of NGB is needed.

Research demonstrated that urodynamic changes, bladder management and anatomical structure changes of bladder and ureter are related to upper urinary tract deterioration of NGB. However, the roles of these factors in detection of UUTD for NGB patients are still controversial, and the interaction of these factors have not been well considered. As a result, current methods are still not precise enough for the risk prediction of UUTD in NGB patients (3–7).

Decision tree is one of the most commonly used data mining techniques. It can establish a set of hierarchical rules from a group of disorderly and irregular cases. Based on these rules, a model can be established to predict the characteristics of a disease effectively with accessible and precise results. It has already been used in various medical fields such as the exploration of risk factors for type 2 diabetes, cancer, and high blood pressure (8–12).

In this research, a decision tree model for the early prediction of neurogenic UUTD was established by analyzing cases of NGB retrospectively.

## Material and Methods

### Patients

A total of 112 patients with NGB were recruited from the Third Hospital affiliated to Sun Yat-Sen University, Nanfang Hospital of Southern Medical University, and Foshan City First People's Hospital (Guangdong, China) between January 2006 and December 2015. This retrospective study was approved by the Nanfang Hospital's ethics committee. Inclusion criteria were that patients should have NGB or secondary neuropathic bladder due to a variety of reasons (ICD-10 code N31.901). Patients were excluded if: 1) they had never accepted imaging tests of the urinary tract; 2) renal insufficiency was not secondary to NGB; 3) had no regular visit to hospital; 4) had no regular urodynamic tests; 5) did not have complete records. Patients were divided into two groups, the case group (n=34) and the control group (n=78), depending on the development of UUTD during hospitalization. UUTD was diagnosed mainly based on the results of imaging examinations, which were found in previous medical records. The diagnosis criteria were: 1) renal insufficiency in diagnosis; 2) results of imaging examinations on urinary tract showed that renal pelvis and/or ureter were full-filled or dilated; and 3) results of imaging examinations on urinary tract showed vesicoureteral reflux (VUR).

### Data collection

As a case-control retrospective study, medical records of patients were reviewed to summarize the history of urinary tract infection, bladder managements, conservative treatments and urodynamic findings. Patients who were diagnosed with urinary tract infection were considered as having history of urinary tract infection. Bladder management methods, including manual micturation by increasing abdominal pressure (like tapping or extruding abdomen), voluntary urination, indwelling urethral catheterization, clean intermittent catheterization and cystostomy, were used to empty bladder in voiding period. Conservative treatments with non-surgical and non-pharmacologic methods, including bladder training, pelvis floor muscle exercises, and pelvis floor electrostimulation, were used to promote rehabilitation of bladder function in voiding period.

All patients accepted the urodynamic tests with Delphis™ Urodynamic Analyzer (94-R01-BT, Canada). In the urodynamic tests, an 8F catheter was first inserted into the bladder through the urethra; then, the bladder was filled with normal saline at 20–30 mL/min in the beginning and gradually up to 50–60 mL/min. The urine samples were collected at the end of filling period. During the filling and voiding periods, patients' bladder sensation and reaction to the tests were recorded. In this study, detrusor activity, bladder sensation, bladder capacity, bladder compliance, urethral function, maximum bladder capacity, Pves max (maximum intravesical pressure), Pabd max (maximum abdominal pressure), and Pdet max (maximum detrusor pressure) were recorded in the filling phase. Detrusor contractility, urethral function, and Pdet max were recorded in the voiding phase during the urodynamic tests. In all cases, urodynamic test results acquired at the onset of UUTD and at the last hospital visit for the case and control groups, respectively, were recorded.

### Statistical analysis

Data analyses were performed using SPSS software (version 22.0, IBM, USA). The *t*-test and chi-square test were used to assess differences for each indicator between the case and control group. Data are reported as the frequency, proportion, and means±SD.

Classification and regression tree (CART) method of decision was applied to develop the model. UUTD was used as the dependent variable and history of urinary tract infections, bladder management, conservative treatment, and urodynamic findings were used as the independent variables. Cross validation was used to verify the data processing results. The regression tree model was built using the standard CRT algorithm in SPSS. The maximum tree depth and surrogates were automatically set by the software. The minimum number of cases to determine parent node and child node were 10 and 5, respectively. Gini impurity was used for impurity measurement and the minimum change in improvement was set as the default value. Tree pruning was not performed.

Sensitivity, specificity, positive predictive value, negative predictive value, accuracy and area under curve (AUC) were used to describe the predictive value of the decision tree model. The Kappa consistency analysis was applied to test the results. A P value less than 0.05 was considered statistically significant.

## Results

The study population consisted of 112 patients with NGB. Of those, 34 had spinal injury, 32 had diabetes, 20 had cerebrovascular accident, 6 had lumbar disc herniation, 4 had spinal pelvic surgery, 2 had craniocerebral injury, 2 had spinal stenosis, 1 had Green-Barre syndrome, 1 had multiple sclerosis, 1 had viral encephalitis, 1 had Parkinson disease, and 8 with unknown cause. The case and control groups were created according to presence or absence of UUTD, respectively (Table 1).

**Table 1.** Baseline characteristics of neurogenic bladder patients (NGB) with upper urinary tract damage (case group) or without it (control group).

Characteristics	Case group (n=34)	Control group (n=78)	P
Age (years)	49.9±18.6	45.2±14.6	0.15
Gender (n, %)			0.205
Male	17 (50%)	49 (62.8%)	
Female	17 (50%)	29 (37.2%)	
NGB duration (days)	7.5 (10.5)	15 (604.25)	0.023
Urinary tract infection (n, %)			0.005
Yes	13 (38.2%)	52 (66.7%)	
No	21 (61.8%)	26 (33.3%)	
Conservative treatment (n, %)			0.005
No	21 (61.8%)	26 (33.3%)	
Bladder function/pelvic muscle exercise/pelvic electric	13 (38.2%)	52 (66.7%)	
stimulation therapy			
Bladder management (n, %)			0.106
Urinate	9 (26.4)	18 (23)	
Indwelling catheter/cystostomy	18 (52.9%)	54 (69.2%)	
Clean intermittent catheterization	7 (20.6%)	6 (7.7%)	
Detrusor activity (n, %)			0.016
Normal	21 (61.8%)	29 (37.2%)	
Overactivity	13 (38.2%)	49 (62.8%)	
Bladder sensation (n, %)			0.004
Normal	12 (35.3%)	11 (14.1%)	
Increased	10 (29.4%)	14 (17.9%)	
Reduced/Absent	12 (35.3%)	53 (67.9%)	
Bladder capacity (n, %)			0.004
Normal	12 (35.3%)	8 (10.3%)	
High	10 (29.4%)	24 (30.8%)	
Low	12 (35.3%)	46 (59%)	
Urethra function (n, %)			<0.001
Normal	34 (100%)	40 (51.3%)	
Incompetent	0 (0%)	38 (48.7%)	
Detrusor contractility (n, %)			0.014
Normal	7 (20.6%)	10 (12.8%)	
Underactive	9 (26.5%)	44 (56.4%)	
Acontractile	18 (52.9%)	24 (30.8%)	
Bladder outlet obstruction (n, %)			<0.001
No	27 (79.4%)	34 (43.6%)	
Yes	7 (20.6%)	44 (56.4%)	
Bladder compliance (n, %)			0.002
≥20 cmH_2_O	15 (44.1%)	58 (74.4%)	
<20 cmH_2_O	19 (55.9%)	20 (25.6%)	
Maximal bladder capacity (n, %)			0.043
≥600 cmH_2_O	10 (29.4%)	39 (50%)	
<600 cmH_2_O	24 (70.6%)	39 (50%)	
Pdet max in filling phase (n, %)			<0.001
≤40 cmH_2_O	22 (64.7%)	15 (19.2%)	
>40 cmH_2_O	12 (35.3%)	63 (80.8%)	
Micturitional Pdet max (n, %)			<0.001
≤40 cmH_2_O	16 (47.1%)	11 (14.1%)	
>40 cmH_2_O	18 (52.9%)	67 (85.9%)	
Pves max in filling phase	74.14±31.45	74.46±35.29	0.964
Pabd max in filling phase	26.08±11.67	20.57±13.13	0.037

Data are reported as means±SD or number and percentages. Pdet max: maximum detrusor pressure; Pabd max: maximum abdominal pressure; Pves max: maximum intravesical pressure. Data were analyzed with the *t*-test or chi-square test.

**Figure 1.**
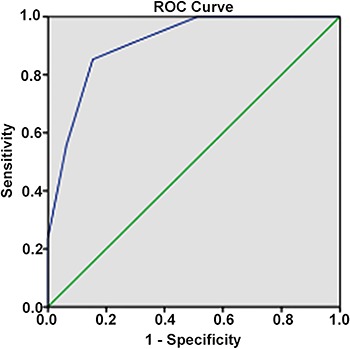
Decision tree model for risk of upper urinary tract damage (UUTD) in patients with neurogenic bladder. Pabd max: maximum abdominal pressure (>14 cmH_2_O); Pves max (maximum intravesical pressure, ≤89 cmH_2_O),

A decision tree model was generated to analyze several indicators for risk of UUTD. Urethra function, Pabd max, Pves max, and gender were selected as the screening indicators, and cut-off values for Pabd max and Pves max were 14 and 89, respectively (Figure 1). Extraction of the higher risk of neurogenic UUTD established in this study indicated two classification rules, one is normal urethra function, Pabd max >14 cmH_2_O, and Pves max ≤89 cmH_2_O, and the other is normal urethra function, Pabd max >14 cmH_2_O, Pves max >89 cmH_2_O and female gender (Table 2).

**Table 2.** Classification rules of the decision tree model for risk of upper urinary tract damage (UUTD).

Rules	Conditions				Risk of UUTD	n
	Urethra function	Pabd max	Pves max	Gender		
1	Normal	>14	≤89	-	79.2%	19
2	Normal	>14	>89	Female	58.8%	10

Pabd max: maximum abdominal pressure; Pves max: maximum intravesical pressure.

**Figure 2.**
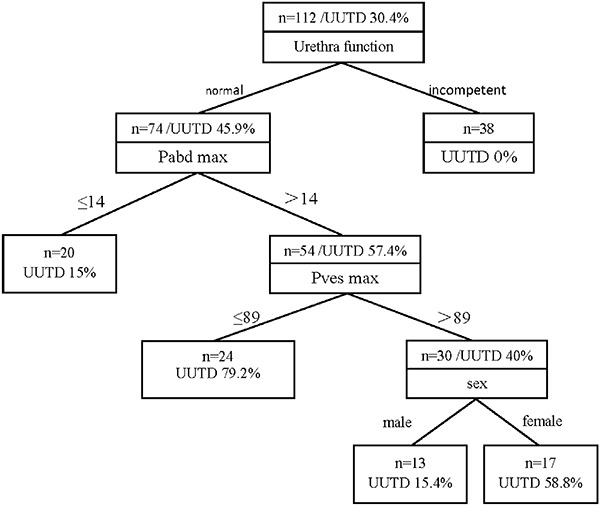
Receiver operating characteristic analysis of the model, where the ROC curve is displayed in blue, and the reference is displayed in green.

The evaluation indices for the risk of UUTD using the decision tree are illustrated in Table 3. The sensitivity, specificity, and overall accuracy in predicting risk of UUTD were 85.3, 84.6, and 84.8%, respectively. In addition, results showed that the AUC was 0.901 (95%CI=0.844–0.958) (Table 3, Figure 2).

**Table 3.** Results of the decision tree model evaluation.

	Evaluation measures					
	AUC	Sensitivity	Specificity	PV+	PV-	Accuracy
Results	0.901	85.3%	84.6%	70.7%	92.9%	84.8%

AUC: area under the curve; PV+: positive predictive value; PV-: negative predictive value.

The accuracy of prediction was measured by the cross-validation method, including risk assessment and its standard error. The risk assessment of this model was 23.2%, which is the percentage of cases that would be wrongly classified (Table 4). Kappa test was used to analyze the consistency of the predicted results. The Kappa value was 0.661 (P<0.001), showing a good fitness between the prediction results and the actual morbidity of UUTD.

**Table 4.** Result of cross validation.

Method	Estimate	Standard error
Resubstitution	0.152	0.034
Cross-Validation	0.232	0.040

## Discussion

UUTD in NGB includes a series of complications such as pyeloureterectasis, hydronephrosis, VUR and renal insufficiency (13). It is demonstrated that the anatomic structure and physiological function of intramural ureter, bladder triangle and ureter undertake the responsibility for anti-reflux. Urodynamic changes, bladder management model, complications of urinary system and some other factors may impair the anti-reflux mechanism and induce the urine in bladder to flow back into ureter. The refluxed urine increases the pressure of renal pelvis and calyces, expanding them, and finally leading to chronic renal failure (14). Until now, most research focused on the influence of a single factor on the UUTD in NGB; few studies are on the risk prediction of neurogenic UUTD.

Decision tree technique, which could establish a set of classification rules from a group of disorder and irregular cases, is a commonly used data mining method in clinical research. Technically, a tree diagram is used to show all the possible outcomes and classify the study objects accurately. Recently, it has been used to excavate major risk factors in some diseases (8–12). The present study established a decision tree model to excavate the major risk factors for UUTD in NGB patients. The results indicated that urethra function in the filling phase was a major predictive variable; also, Pabd max, Pves max, and gender were associated with the occurrence of the UUTD in NGB patients.

Under normal urethra function, urethral closure pressure can still maintain a positive value, which would induce urinary continence during filling phase even with increased abdominal pressure. However, if urethra function is incompetent, urine leaks out and increases abdominal pressure even if there is no detrusor contractility. In patients, the injured micturition reflex nerve would lead to the inactive sphincter contractility during bladder filling phase or the abnormal urethral closure pressure, both of which would lead to an incompetent urethra function. The leakage of urine from urethra could decrease Pves in filling phase and reduce the reflux of urine from bladder to ureter. Further, the incompetent urethra function would induce leakage of urine from urethra, which means the reflux of urine to urethra is decreased. There were 45.9% of patients with UUTD showing normal urethra function, while none of the patients showing incompetent urethra function was with UUTD. The results indicated that urethra function during filling phase is a major predictive variable for risk of UUTD in NGB patients and the root node of the decision tree.

In our study, Pabd max and Pves max were valuable predictive variables for risk of UUTD in NGB patients. In normal conditions, under the effect of Pabd, a certain Pves in bladder is determined during the filling phase. When Pves in bladder is raised to a certain degree, micturition reflex is induced to maintain a lower pressure in bladder. As long as the intravesical pressure is in a safe range (less than 40 cmH_2_O) in the filling and voiding phases, urine does not reflux into ureter. If the intravesical pressure increases excessively, there is difficulty for urine in upper urinary tract to be delivered from ureter to bladder; then, the persistent high intravesical pressure would lead to UUTD (2,15–18). The present study showed that 57.4% of patients with Pabd max >14 cmH_2_O were with UUTD, while 15% of patients with Pabd max ≤14 cmH_2_O were with UUTD, suggesting that high Pabd would increase the risk of developing neurogenic UUTD. In this study, there were 78.6% of patients with Pves max >40 cmH_2_O. The results indicated that UUTD occurred in 79.2% of patients with Pves max ≤89 cmH_2_O, and among them there were many patients with Pves max >40 cmH_2_O. Although some patients' Pves max were less than 89 cmH_2_O, the mean value of Pves max in each group was much higher than 40 cmH_2_O, which might indicate increased risk of neurogenic UUTD. The results also showed only 40% of patients with Pves max >89 cmH_2_O developed UUTD. This indicates that the risk of UUTD gradually increases until the Pves reaches a certain degree and then the risk does not further increase when the Pves exceeds 89 cmH_2_O. The mean value of Pves max in each group was much higher than 40 cmH_2_O; however, because the sample size of our study was limited, it was quite difficult to find the exact cutoff point of Pves max to classify the risk of developing UUTD accurately. Further research and discussions with a larger sample size are needed.

The present study showed that gender is also a predictive variable for risk of UUTD in NGB patients. Because of different anatomical structures, women suffer more easily from urinary tract infection and high detrusor pressure, and the morbidity of UUTD in women may be higher than in men (3). The study revealed that gender might be a node in the decision tree, indicating that females had higher risks of neurogenic UUTD than males.

The decision tree model for risk of neurogenic UUTD established in this study summarized two classification rules. One is normal urethra function, Pabd max >14 cmH_2_O, and Pves max ≤89 cmH_2_O, and the other is normal urethra function, Pabd max >14 cmH_2_O, Pves max >89 cmH_2_O and female gender. NGB patients in accordance with the classification rules may be at a higher risk of developing UUTD.

Our study developed a decision tree model to predict the risk of UUTD in patients with NGB. Further, it might provide a new and convenient way to screen UUTD in NGB patients in undeveloped and developing areas.
